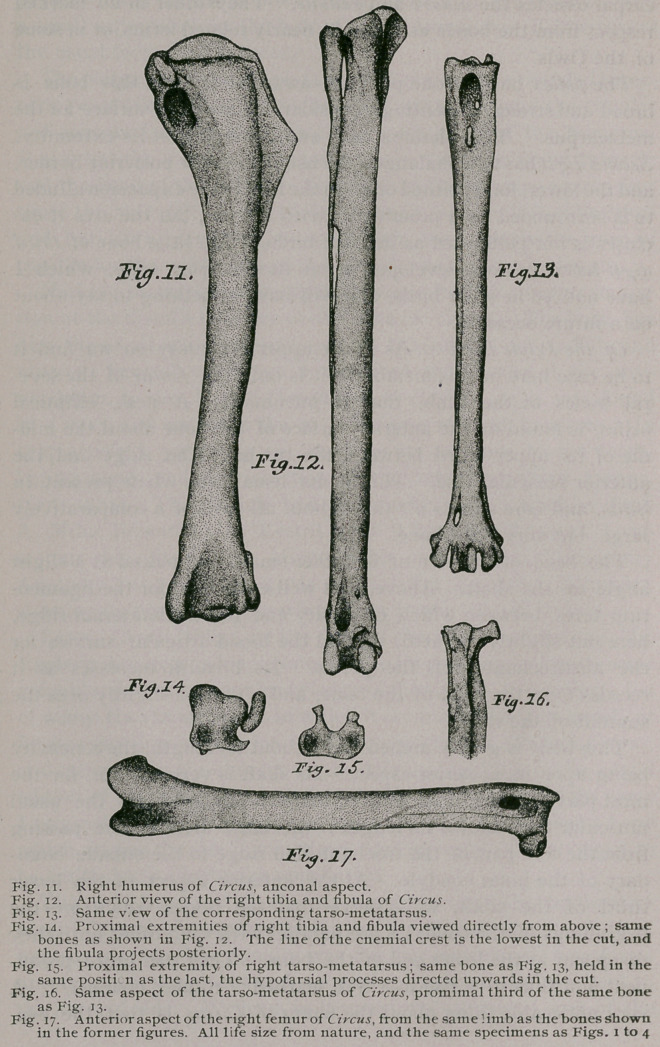# Osteology of the Circus Hudsonius

**Published:** 1889-04

**Authors:** R. W. Shufeldt


### ART. X.—OSTEOLOGY OF CIRCUS HUDSONIUS.

BY R. W. SHUFELDT, M. D., C. M. Z. S.

The object of the present memoir is simply to present a detailed account of the osteology of a good representative American Hawk. For several years past I have been collecting material with the view of monographing the subject of the osteology of North American Falcones, but at the present time this material is not quite sufficiently extensive to undertake the work in the way I have hoped to complete it. Several years ago I published monographs devoted to detailed descriptions of the skeletons of *Speotyto cunicularia hypogæa*, and the Family *Cathartidæ*, so the principal part still left undone in this field, for our birds of prey, is among the Hawks, Eagles, Kites, the Osprey, Falcons, and some of the Owls.

*Of the Skull.*—In dealing with this part, as well as the remainder of the skeleton of *Circus*, I will take into consideration only the skull of the adult individual; making no attempt to give exact definitions of the boundaries of the several elements of the skull, a thing which is only possible in immature specimens.

We observe upon lateral view (Fig. 1) of the skull of this Hawk that the *premaxillary* is produced downwards anteriorly into a sharp-pointed hook. The upper boundary of this, strongly convex, forms a little less than half of the culmen, commencing as it does at the apex of the osseous beak, and extending back to where the nasal processes of the bone commence. Here the premaxillary presents another convexity as it passes over the nostril to gradually terminate, where its nasal processes articulate with the frontals in the median line. The opposite or posterior margin of the hook above mentioned, is likewise convex anteriorly, and its margin is produced backwards, forming the border of the dentary process of the premaxillary, it again becomes convex from above downwards. This latter convexity forms quite a perceptible swell in the bone, just before it receives the insertion of the maxillary. The osseous nostril is elliptical in outline, and these two opposite apertures are separated from each other to the extent shown in the figure, by an osseous nasal septum. This septum has a transverse partition, joining, but not rising above the middle of the nasals, and merging into the above mentioned longitudinal one, which latter is then produced backwards nearly to meet the ethmoid, while anteriorly it gradually slopes downwards and forwards by a gentle convexity to merge into the margin of the anterior third of the osseous nostril.

As is the case in nearly all birds, the posterior boundary of this nostril is formed by the *nasal*, which bone in the present subject, has become thoroughly incorporated so far as its sutural borders are concerned, with the other elements with which it comes in contact, with the exception of the nasal process of the premaxillary (Fig. 2). We are likewise enabled to see upon lateral view the extensive *maxillo-palatines* of this Hawk. These very delicate bones are of a highly spongy texture here, and rise up nearly as high as the ethmoid. Anteriorly they attach themselves both to the nasals and the inter-nasal septum. As they are produced backwards they lie nearly parallel to each other, an interspace existing of about two millimetres into which the vomer extends in the median plane. Below, their tissue is a little denser, their borders are rounded, while they merge into each other anteriorly on this aspect with the palatines and premaxillary (Fig. 3). Their union with each maxillary is through a horizontal plate, which is not perforated by any foramina. The *lacrymal* of *Circus* is quite a large bone, as it is in most Hawks. It articulates with the frontal alone, on an extensive facet situated on the extreme anterior and outer margin of that bone, just where it is overlapped by the nasal. From this point the lacrymal throws out, horizontally, being at the same time directed somewhat backwards, a broad "superciliary process" (Fig. 2), while it sends downwards a flattened and much smaller process, concave in front, convex posteriorly, which touches by its apex the maxillary bar (Fig. 1).

At the postero-external margin of either lacrymal there is always to be found a free "accessory piece" consisting of a small osseous scale, horizontally attached to the bone by a semiligamentous tissue.

The lacrymal of *Circus* is a thoroughly pneumatic bone, and presents for examination several confluent foramina, which open on its inner aspect at the junction of the supercillary and desending processes.

The anterior border of the superior half of the *ethmoid* is broad, flat, and somewhat thickened ; and this part of the bone reaches forward beyond the aliethmoid plates, to form a substantial base upon which the frontals and premaxillary rest. Anteriorly, the lower margin of the ethmoid is sharp where it joins with the rostrum.

The aliethmoid plate is conspicuous on lateral aspect of the skull. Its posterior surface looks upwards, backwards and outwards, the plane being reversed for the anterior surface. In outline it is an oblong plate, which is quite true for its lower and free end, while the opposite end is broader and merges with the mesethmoid. At its superior and inner angle, just beneath the frontal, it is pierced by an elliptical foramen for the passage of the olfactory nerve. Beyond, it develops a small bony canal for the further protection of this branch. The interorbital septum presents near its middle one large, elliptical vacuity, with the major axis of the ellipse about parallel with the zygomatic bar.

In the recess of the angle between this septum and the frontal bone, we find the double groove for the lodgment of the olfactory nerve, the grooves commencing directly in front of the olfactory foramen, running parallel with each other quite up to the opening for their passage through the aliethmoidal plate. The zygomatic or jugal bar is very slender in *Circus*, and the sutures of its original elements are quite obliterated. Its quadrate end develops at right angles a peg-like process, to articulate in a corresponding pitlet in that bone. The maxillary or anterior extremity has already been sufficiently described. Its relations with the palatines and maxillo-palatines are well shown in Fig. 3.

The superior margin of the orbit is rounded, but as this proceeds backwards it soon becomes sharp, a condition it retains to the very tip of the sphenotic process.

At the back of the orbit the wall is broad and gently concave throughout ; it being pierced at its lower and inner angle by a circular optic foramen, and the foramina more external to it are quite distinct from each other, which is by no means the rule generally among birds.

The outline of the olfactory foramen, leading into the brain case is very irregular, and the wall in its immediate neighborhood is thinned to the extent of perforation in one specimen before me, while in another two minute formina occur, just large enough, on either side, to admit the passage of the nerves, and the aforesaid perforation is much smaller. Quite an extensive osseous flap is thrown out to shield the opening to the ear behind. This latter aperture is comparatively very large, the opening being fully equal in size to the corresponding one in a specimen of *Falco r. gyrfalco* from Alaska, which I find in my collection, and, as we know, a very much larger Hawk than *Circus.* We have to account for this, in the well-known fact, however, that in the structure of the ear parts the Marsh Harrier approaches the Owls. In the upper part of the recess, formed by this aural cavity, the double head of the *quadrate* articulates, the outer head with the squamosal, the inner one with the bony wall within. This bone then becomes twisted on itself, to support below the usual articular facet for the mandible, which facet is quite narrow from before backwards, and rather long transversely. It presents two articular surfaces, an outer and an inner, connected by a narrow isthmus posteriorly, and separated by a shallow concavity anteriorly.

The quadrate throws inwards a stumpy orbital process, the anterior surface of which lies in the same plane with the general anterior surface of the bone, it being directed upwards, forwards and outwards. On the posterior surface of the quadrate we find a longitudinal depression coming down from between the two heads mentioned above, which harbors one of the pneumatic foramen, the other being found at the base of the orbital process on this aspect. The peculiar form of the cranial vault with the bulging supra-occipital prominence, should be noted on this lateral aspect of the skull.

Upon a superior view of the skull of the *Circus* (Fig. 2), the principal points to be observed are, among others, the position of the elliptical, osseous nares; the direction of the cranio-facial suture, which is not in this Hawk drawn directly across in a transverse line, as it is in *Falco sparverius* for instance. It is to be observed also that the sutural traces of the nasal processes of the pre-maxillary are quite distinct in adult skulls, while in some Falcons they are entirely obliterated. The distance between the

Fig. 1. Left lateral view of the skull and mandible of *Circus hudsonius*.

Fig. 2. The same specimen from above; mandible removed.

Fig. 3. The same seen from below; mandible removed.

Fig. 4. The hyoid arches from below. All these figures are life size from nature, from an adult female subject collected by the author in Wyoming (1878).

superorbital margins is very narrow, and a shallow, longitudinal, median groove courses between them, nearly as far in a backward direction as the supra-occipital prominence. The parietal eminences are smooth and somewhat prominent. Venous grooves are seen running over them and leading to minute foramina just within the orbital margins.

The superciliary processes of the lacrymals are well seen upon this view, and it is to be noticed that their outer extremities support "accessory pieces," as in some other falconine forms; moreover, these bones are very loosely articulated with the frontals on either side, and they are sure to come away in the course of ordinary maceration. From above we can also see the aliethmoids and the palatines extending back of them, as well as the anterior margins of the quadrates with the zygomatic bars leading from them. The maxillaries show also upon this view, just beyond the lacrymals.

One of the most striking features upon basal view of the skull of *Circus* is, how all the bones lie nearly in the same horizontal plane, this plane extending from the posterior margin of the foramen magnum to the descending hook-like process of the beak formed by the premaxillary. This feature is quite characteristic of some of the other genera, but not to the extent as seen in this Harrier.

Just within the point of the beak are four small foramina, arranged as if they occupied the angles of a square. These openings are seen in other Hawks and Falcons. Immediately behind them we see in *Circus* the space where the palatines and maxillo-palatines merge into the premaxillary. On either side, and external to this, is a foramen formed by the bones surrounding it—the maxillary, the palatine and the dentary process of the premaxillary.

The major part of the *palatines* lie in the horizontal plane; they are broad behind, where they are marked on their inferior surfaces with shallow depressions, to run out into narrow bars anteriorly. The interpalatal space is broad, being fully three millimetres across its narrowest part. In this space we see the vomer and the maxillo-palatines. A small part of the palatines posteriorly curve upwards, affording by their firmly united superior surface a concave groove to ride upon the rounded surface offered by the anterior half of the rostrum, while beyond this they anchylose in the median line with the vomer (Fig. 3). The articular heads of the palatines also rest upon the rostrum, side by side, with their facets looking almost directly backwards to articulate with the pterygoids.

The *vomer* (Fig. 5) can best be studied in a longitudinal and vertical section of the skull, passing very slightly to one side of the median line. This I have been enabled to perform on one skull by means of an exceeding fine jeweller's saw. The appearance upon the cut side of such a section is well seen in the figure referred to, where the position of the vomer, there marked *v*, can be easily observed. It is seen to be a thin lamina of bone, flattened from side to side, and shaped much like a long *ſ*. Its anchylosis with the united palatines seems to be complete, while its anterior extremity is pointed and free. The maxillo-palatines have already been fully described above, their relation to the inter-nasal septum and the vomer can also be seen in Fig. 5.

Fig. 5. A vertical, longitudinal section of the skull of *Circus*, made nearly in the median plane, designed to show the capacity of the brain case, and the position of the vomer, *v*. Life size by the author from his own dissections.

The *pterygoids* are a very slender pair of bones in *Circus;* anteriorly they articulate with the palatines and the rostrum of the sphenoid, although they fail to come in contact with each other at this point. Their posterior extremities are expanded and cup-shaped to allow them to articulate with a corresponding convexity on each quadrate. They do not meet the basi-sphenoid by articulation with basi-pterygoid processes developed on the part of that latter bone, as we see them in the owls (*Surnia*, *Speotyto*, and others). At the points, however, where such processes are developed, *Circus* possesses a sharp-pointed spicula of bone on either side, and this is opposite a corresponding enlarged part of each pterygoid (Fig. 3). These two projections are separated from each other by at least two millimetres in life, *i. e.*, the pointed rudimentary basi-pterygoid process and the enlargement on the corresponding pterygoid.

The basi-temporal and basi-occipital regions are well depressed (viewing the skull in the position of Fig. 3) below the exoccipital regions and other surrounding parts. A thin lip of bone overhangs the openings, which are here separate Eustachian, tubes; while the formina for the internal carotids lay to their outer sides, posterior to them and just above the anterior tympanic recess. The foramina for the exit of the other cranial nerves that issue from the brain-case occupy their usual sites and offer nothing peculiar for description. They agree with Parker's figure of the nestling *Accipiter nisus*.

The condyle is hemispheroidal in form, and very small; it barely encroaches upon the periphery of the foramen magnum. This latter aperture is nearly round, and lies quite in the plane of the basi cranii. This condition seems to be characteristic of the *Falconidæ*.

A posterior view of the skull of *Circus* presents a smooth, semi-globular surface. At its lower part, in the median line, we observe a well-developed supraoccipital prominence, with a decided concavity on either side of it. On this view we are just enabled to see the condyle, and only the outer projections of the quadrates. Laterally, the squamo-exoccipital wings hide other things from view beyond. Above these wings the sphenotic processes hang down. The shallow median groove passes between the parietal eminences. In the brain-case we observe (Fig. 5) that the carotid openings are separate, being some distance apart in the pituitary space.

The wall covering the anterior semicircular canal is much raised, while beyond it the usual group of foramina for the exit of the seventh (the vagus) trifacial division of the fifth and other nerves are seen.

The fossæ for the lodgment of the several encephalic lobes are very deep, and this condition is heightened by an ossification of the tentorium, which divides them, for some little distance beyond the inner cranial wall along the site of the attachment of that membrane. The optic nerves make their exit at separate openings, already alluded to above.

The greatest amount of diplöic tissue is found between the inner and outer cranial tablets, at the vault of the cavity, or that portion covered by the frontal bones, as it is in these latter that it exists. In the superoccipital region it is quite scanty, and the cranial walls are here very thin (Fig. 3).

Many of the bones in the skull of this Hawk are pneumatic, this part of the skeleton when dried weighing but thirty-eight grains (Troy), and this includes the lower jaw.

The *mandible* may be said to partake of the V-shaped variety, although its inner outline borders closely on the U (Figs. 1 and 6). The symphysis is gently curved down anteriorly so as to look upwards and forwards. Each ramus has rounded superior and inferior borders, and their width is quite uniform from the coronoid process to symphysis on either side (Fig. 1).

Upon this aspect, too, we observe that the ramal vacuity, seen in so many birds, indeed in other Hawks (*Falco*), has here been entirely absorbed. Every evidence of original sutural landmarks has been obliterated, and the mandible of this Hawk is as good an example as we will find anywhere among the class of a "single bone." One not acquainted with its composition in the nestling would never suspect anything else after a careful examination. The in-turned tips of either articular end is at right angles to the median plane. Each presents an elliptical pneumatic foramen just within the tip. Concave articular facets are seen, which correspond to the convex surfaces, as described on the foot of each quadrate. There is a rudimentary "posterior articular process" present. The coronoid process, on either ramus, is but feebly developed and only slightly elevated above the general line (Fig. 1). When articulated with the skull the superior line of the ramus ceases to be approximated to the osseous superior mandible at a point on the middle of the dentary process of that bone. From this point it curves gently downwards until at the tips of each mandible they are four millimetres apart. This condition is seen in the *Cathartidæ* also. (See author's "Osteology of the Cathartidæ," Figs. 105, 106, 115 and 116.)

Fig. 6. Mandible of *Circus* seen from above. Life size from nature.

On the hyoid arches we find that the glosso-hyal remains in cartilage throughout life (Fig. 4). The cerato-hyals or "lesser cornua" are quite individualized, being simply connected by a transverse bar at their middles, affording the articular facet for the basi-hyal. This letter element is co-ossified with the basi-branchial or uro-hyal, the two bones forming one piece in the adult Hawk. The cerato and epibranchial elements are up-curved, slender, cylindrical rods of bone, the latter being slightly tipped on their posterior extremities with cartilage. No doubt but that the uro-hyal is also tipped with this material, and it has been lost in my specimens, as I see such is the case in the Falcons, (*Falco r. gyrfalco*).

*Circus* presents the desmognathous type of structure so far as its palate is concerned, and falls within the group *Aëtomorphæ* of Huxley.

Not having a nestling of this Hawk at hand I am unable to say definitely whether the "median septo-maxillary" ossifies as a distinct element or not,—that it is present in the adult there is no doubt. The desmognathism in *Circus*, and the union of its maxillo-palatines with the nasal septum takes place beyond the broad processes thrown off by the maxillaries, while the spongy part of the maxillo-palatines are produced far backwards with a narrow valley between them.

This arrangement is very different in *Falco*, where the fusion of the maxillo-palatines is entirely opposite the maxillary processes, if anything somewhat more posterior to them, and, after their separation, the intervening valley is much wider.

I have failed to detect in *Circus* either a medio-palatine or the meso-pterygoids, that is, as I interpret these elements as they have been described by others. To still further pursue our recapitulation of the characters presented in the skull of *Circus*, it is to be inferred from the presence of the rudimentary basi-pterygoidal processes of the sphenoid, that this Harrier, (the condition being an approach towards the more embryonic types,) stands among the lowest of the birds of prey. There is no evidence of the presence of these processes in the skull of *Falco r. gyrfalco*. I would say then (were we to judge from this character alone), that this Falcon stood higher in the scale of organization than *Circus* does. The style of the vomer has been sufficiently dwelt upon. The inner condyle of the quadrate is lower than the outer, and at the same time the smaller of the two.

Parker tells us that "in the Sparrow-Hawk distinct pterotic and sphenotic centres are developed ; and the orbito-sphenoids are preceded by cartilage." ("Morph. of the Skull," p. 264).

*On the Axial Skeleton* (Figs. 7–10).—The cup for the occipital condyle on the anterior aspect of the *atlas* of *Circus* presents a distinct notch in its superior periphery. Above it, the neural canal is a transverse ellipse, the neural arch closing it superiorly being quite broad. Below, two short processes are directed backwards behind the part bearing the articular cup.

The "odontoid process" of the *axis* is compressed from above downwards, its surface being flat superiorly, convex below. The neural canal is circular, and the arch above supports three stumpy processes, the lateral diapophyses and the neural spine. Beneath the odontoid process the atlantal articular surface is a shallow concave ellipse, placed transversely. Behind this the body of the bone is compressed from side to side, with longitudinal median crest, terminating posteriorly in a knot-like process.

The *third vetebra* presents pre- and postzygapophyses ; the articular facets on the first being directed upwards, those behind directly downwards. These processes in this vertebra are united by a horizontal plate of bone, which lends to this segment a very solid appearance not possessed by those behind it. It is pierced about the middle on either side, near the outer margin, by a minute foramen. A median neural spine projects backwards from the posterior border.

The neural canal is cylindrical, and the arch slightly overhangs it behind, but recedes from its anteriorly. On either side the vertebral canal is present a minute perforation ; the parapophyses having short spiculæ directed backwards. A median, oblong hypapophysis is situated posteriorly directly above which is the articular facet for the fourth vertebra. It is concave from above downwards, and convex from side to side, the reverse being the case in the anterior facet, which is directed downwards and slightly forwards.

In the *fourth vertebra* the pre- and postzygapophyses are connected by a delicate spine on either side, the articular surfaces on the former are slightly inclined towards the median plane and each other, the reverse being the case on the latter. The neural spine is more stumpy and has worked towards the middle of the arch ; the canal is smaller and still circular ; while the vertebral canals are larger, longer, and their lateral wall is perforated on on either side by a small foramen. The parapophysial spines extend backward as far as the posterior articular facet, and the hypapophysis is in the middle of the body of the vertebra.

In the *fifth vertebra* the neural spine still maintains its position as in the last segment, but is rapidly disappearing. The facing of the articular surfaces on the zygapophysial processes is more decided, while those on the posterior pair are borne on projecting and diverging limbs of considerable length. The delicate bar that connected them on either side, in the fourth vertebra is here deficient at the middles. Other features are but slightly modified; the hypapophysis has assumed a position just behind the anterior articular surface of the body.

The neural spine of the *sixth vertebra* is barely perceptible, and the interzygapophysial bar is again intact as a delicate bridge. At the base of each postzygapophysis above, a little projection is seen, which occurs on the four succeeding segments, both then are obliterated.

The vertebral canals have the form of a vertical ellipse, and the parapophysial spines are again shortening. Beneath, we observe that the hypapophysis has disappeared, and at its site, in the last vertebra, the carotid canal begins to form. The body of this vertebra is nearly square on transverse section. But slight modification has taken place in the *seventh vertebra*. The limbs of the postzygapophyses are shorter; the connecting bar is still intact; the neural spine has entirely disappeared; and the carotid canal is deeper and narrower.

In the *eighth vertebra* the interzygapophysial bar is once more incomplete, while the changes taking place in the last vertebra are becoming better marked.

Sharp lateral processes form the walls of the narrow carotid canal in the *ninth vertebra*, and the vertebral canals are nearly circular and increasing in calibre. The parapophysial spines are nearly as long as the body, while the vertebræ are now beginning to be shorter and heavier. The anterior pair of articular facets look upwards and inwards, the reverse being the case with the hinder pair. A tuberous neural spine and hypapophysis make their appearance in the *tenth vertebra*, the latter being in the middle of the body. Parapophysial processes are shorter, though more pronounced, while the carotid canal has ceased to exist. The general form of this vertebra is cubical.

In the *eleventh vertebra* the neural spine is more lofty and hooks forwards; the spine beneath forms a low median crest nearly as long as the body of the vertebra. The vertebral canals are still increasing in calibre. Quite marked changes have gradually come about in the *twelfth vertebra*. The neural spine is very pronounced, while the hypapophysis is shrinking again in importance. In the parapophyses the form of the diminitive rib begins to be suggested, accompanied by a corresponding enlargement of the vertebral canals. On the centrum, the articular facets are larger, and the anterior one, especially, deeper. The neural canal, still circular, is here larger than we found it in the axis. It seems to have the least calibre in the sixth vertebra.

In figure 7, the anterior vertebra shown, is the *thirteenth*, and it departs very markedly from the last one described. Its neural spine now becomes a high quadrate crest nearly as long as the centrum of the bone.

The transverse processes are heavier, and the bases to the zygapophyses very substantial, with little change in the direction of the facets. A rudimentary *free* rib has made its appearance, the body of which is no longer than its neck. I should have noted a pneumatic foramen on the lateral aspect of the centrum of the twefth vertebra ; it is still larger here ; is seen in the fourteenth ; largest of all in the fifteenth ; very minute in the succeeding one ; and disappears in the seventeenth.

The calibre of the neural canal in the thirteenth vertebra is circular and large—it gradually diminishes to the nineteenth, where it is just a little more than half the size.

The centrum of the thirteenth vertebra is broader than it is deep, and this segment is quite short from before, backward. Below, a tricornute hypapophysis is beginning to be developed. In the *fourteenth vertabra* the neural crest is a little longer but no higher ; the transverse processes are still more spreading, while the free pair of ribs are now quite long, though they do not reach the sternum, or rather are not met by costal ribs. They are devoid of epiplural appendages. The centrum is evidently becoming narrower and longer, and this contraction and lengthening gradually continues through the nineteenth or last free vertebra we find before reaching the pelvis, in which the centrum is twice as long as it is wide. The articular facets also increase proportionately in size ; the periphery of the posterior articular facets on the centrum of the nineteenth vertebra is fully double the circularity of its neural canal, the measurement for the latter being taken over the middle of the centrum.

Returning to the fourteenth vertebra, we find the tricornute hypapophysis but little larger than we found it in the thirteenth, but an evident disposition to contract at its base and project into the pleural space. A circular pneumatic foramen is found behind the transverse process on either side from the thirteenth to the

FIG. 7. Right lateral view of a part of the axial skeleton of the *Circus hudsonius*. Showing the shoulder girdle, dorsal division of the vertebral column, the sternum, pelvis, caudal vertebræ, and pygostyle. The parts are all *in situ*. This figure shows well the posterior and detached portion of the pubic element of the pelvis. Same specimen as figured in 1 to 4. Ribs of left side all removed. Life size from nature.

nineteenth vertebra inclusive. This pair of openings are largest in the eighteenth vertebra, and smallest in the thirteenth.

The "intervertebral foramina" become more circular and yet smaller as we proceed toward the hinder part of the spinal column.

In the *fifteenth vertebra* the neural crest interlocks at its posterior superior angle with the anterior superior angle of the neural crest of the sixteenth vertebra, by the arrow-head joint. This interlocking continues throughout the series until we arrive at the pelvis, where no such joint is found to exist. The neural spines or crests through this "dorsal region" of the column become gradually lower and longer as we proceed towards the posterior extremity of the body.

From the fifteenth to the nineteenth vertebra inclusive, the articular facets on the zygapophysial processes gradually change their direction to meet the requirements of the "dorsal region,"—they once more come to face directly upwards anteriorly, while the reverse holds good behind; we observe also that the transverse processes in this series become longer and longer as we proceed in the same direction, and their outer extremities armed in each case with a single, delicate metapophysis which overlaps the process both before and behind it. In the fifteenth vertebra, now under consideration, the hypapophysis loses its tricornute character, and the short pedicle merely supports a circular disc, with its inferior surface directed slightly forwards. This pedicle in the sixteenth becomes longer, and the disc becomes an ellipse, placed longitudinally upon it. The hypapophysis on the seventeenth vertebra dips well down into the pleural cavity as a laterally compressed hook with slightly dilated apex. It is truly claw-shaped in the eighteenth vertebra, though still compressed from side to side, to be entirely absent in the nineteenth.

In *Circus* all the vertebræ are freely articulated upon each other as they are in the owls, from atlas to the one that first anchyloses with the ilia; in *Falco sparverius*, however, from atlas to thirteenth, inclusive, are free, while fourteenth to eighteenth are thoroughly fused into one bone, the outer angles of their diapophyses even being united by anchylosis.

In this common "dorsal" piece of the Sparrow Hawk the two leading vertebræ support hypapophyses. These have also fused together, leaving only a circular foramen between them. The nineteenth vertebra of this little falcon is free and articulates with the posterior one of the consolidated bone in question, and behind with the first one of the pelvis.

The fifteenth vertebra in *Circus* has a pair of true ribs, *i. e.*, they are connected with the sternum through the intervention of costal ribs or hæmapophyses, the two being freely articulated. This pair of pleuropophyses also have unciform appendages, that on either side anchylose on the lower third of the rib, their apices being directed upwards. The facets for the heads of this pair of ribs are upon the anterior margins of the neurapophyses, just above the centrum of the vertebra. This position of these facets obtains for the remainder of the series of articulated pleurapophyses. The facets for the tubercles are at the ends of the diapophyses, and look directly downwards and outwards throughout this region.

The vertebral ribs of the sixteenth to the nineteenth vertebra inclusive become gradually longer as we proceed backwards; they all bear large anchylosed unciform appendages, with their apices directed backwards, of a form shown in Fig. 7. They are laterally compressed and offer large articulatory facets for the costal ribs.

The sternal rib of the fifteenth vertebra, the first of the series, articulates high up on the costal process of the sternum. It is short and straight. As we proceed towards the pelvis we find them becoming gradually longer, flatter from side to side, and more curved upwards, their convexities being below. They articulate with the sternum by extensive transverse facets (Fig. 7).

The two leading vertebra of the pelvis each have a pair of ribs also, that in no way differ from those that I have just described, excepting that the last pair are without unciform processes. They otherwise simply continue the series, and it is evident that the arrangement presents seven pairs of pleurapophyses, which are connected with the sternum through the articulation with an equal number of pairs of hæmapophyses, which in their turn articulate with the costal borders of the sternum by their transverse facets. Both the true and costal ribs of this Hawk are pneumatic.

We will now for a moment leave the vertebral column proper and pass to the consideration of the *sternum* (Figs. 7 and 8). On outline, the general form of this bone in *Circus*, viewed from above, is a parallelogram. Its superior or dorsal surface is deeply concave, accompanied, of course, by a corresponding convexity of the pectoral aspect. The middle of the median line above presents a row of pneumatic foramina leading to the keel. Similar openings occur also on the interfacial spaces on the costal borders; in groups just within the costal borders and the anterior border of the body; and at the bases of triangular pits, one of each, which occupy the inner aspects of the costal processes.

The hinder border of the sternal body is gently concave, and in the specimen in hand the right side is pierced by two foramina,

*Fig. 8.*

*Fig. 9.*

Fig. 8. The sternum of *Circus*, viewed from below. I have specimens before me wherein the sternal foramina are exceedingly small, and I should not be at all surprised to find a sternum of this Hawk lacking these foramina entirely.

Fig. 9. Heads of scapula, clavicle and coracoid, inner aspect, showing their mutual relations. *s.* scapula; *cl.* clavicle; and *c.* coracoid. Same specimen as Fig. 7. Life size from nature.

while only one occurs on the left, as shown in Fig. 8. The sternum of *Falco sparverius* has a large elliptical foramen on either side, whose peripheries so far encroach upon the posterior margin of the sternal body as to slightly absorb it at the point of tangency. In *Falco richardsonii* these foramina are well within this border.

But one pair of muscular lines presents itself upon the otherwise smooth ventral surface of the sternum of *Circus*. Two of the lines are seen on each side of the keel (Fig. 7). The carinal angle is rounded, the anterior border of the keel being concave, while the inferior one presents a graceful convex curve. Posteriorly it terminates at the apex of a triangular smooth surface, the outer basal angles of which are opposite the foramina in the xiphoidal extremity (Fig. 8). The line of union between keel and body is rounded, being concave outwards.

Marked differences occur in the manubrium of Hawks; here in *Circus* it is a stumpy process, generally inclined upwards, having a sharp median edge below and a triangular anterior surface. Among the *Falcons* (*Falco richardsonii*), it is a narrow spicula of bone, directed forwards and upwards; but what is most singular, there exists in these birds a second process that springs in the median line from the border of the body above. These two processes have the coracoidal grooves between them.

The grooves for the coracoids decussate in *Circus*, their inner ends terminating in points—they decussate still more in *Falco*, where their inner ends are rounded. Such a decussation of the coracoidal beds in likewise to be seen in the Herons, as in the genus *Ardea*.

In the specimens of all the *Falconidæ* before me, it is the *right* coracoidal groove that is the anterior one, and overlaps the superior surface of the base of the manubrium. As well as I can remember such is also the case with the Herons.

Returning now to the spinal column, we find that the *twentieth vertebra* of *Circus* becomes anchylosed beneath the ilia. Its broad neural spine has fused into one piece in common with the others that extend back as far as the sacrum; its diapophyses are half covered by the anterior iliac borders, and these with the next vertebra behind show the facets for the two pair of ribs already described above, which are here over-arched by the ilia (Fig. 7).

The anterior aspect of the twentieth vertebra presents all the requirements for articulation with the one next beyond, in its prezygapophyses, and in its centrum. Metapophysial spines however, are only thrown back by the segment before it, while the locking of the neural spines does not take place.

This description of the twentieth vertebra brings us to a point where we must needs take into consideration the *pelvis* of *Circus*. (Figs. 7 and 10). Upon superior view of this bone (Fig. 10) we observe that the neural spine or rather its upper surface, projects forwards as a broad process between the ilia, and is roundly notched anteriorily. The common top of this neural spine for nearly the entire length of the pelvis is smooth, and presents little or nothing to indicate where the divisions among the vertebræ take place. The parts of the last vertebra, that became anchylosed with the pelvis, are easily made out. Very minute inter-diapophysial foramina may pierce this region ; others are but indicated by minute dots. Along the mid-region, the ilia rise above these fused vertebræ in sharp crests, which crests in being produced backwards form the outer margins of these pelvic bones where they constitute the postacetabular surface.

The "ilio-neural grooves" are closed in, but they exist as capacious "ilio-neural canals" beneath the ilia anteriorly.

Each ilium has a rounded anterior border, which presents a slightly raised emargination just within it.

The pre-acetabular surface of the ilia is fully twice as long as the post-acetabular, and its superficies is also double in extent. (Fig. 10). In each bone the former surface, anteriorly, is first directed upwards and only slightly outwards, as it passes backwards it faces almost directly outwards, a direction which it maintains for the rest of its extent. The post-acetabular surfaces of the ilia are confined to two elliptical areas, which roof over the ischiadic foramen on either side, and the direction of whose surfaces is upwards. Upon lateral view of the pelvis (Fig. 7), we see a circular acetabulum with a very deficient base,—the periphery of the inner circle being but little smaller than the outer rim of the cavity.

The anti-trochanter is long and narrow. The plane of the ischiadic foramen is directed downwards, backwards and outwards, and this aperture is completely ovsrshadowed by the ilium. The ischiadic area is generally concave and triangular, the apex of the latter being directed backwards.

Considerable interest attaches to the condition in which we find the pubic bones in *Circus*. The anterior limit of one of these bones, after it leaves the acetabulum, closes in the obturator foramen quite completely, but does not pass beyond. Then occurs an interval, below the lower margin of the ischium, which in life is filled in by ligament, that connects the floating part of the remainder of the pubic bone behind. This latter piece is simply suspended from beneath the posterior angle of the ischium by ligament, not in any way connected with the anterior limb of the pubic rod, except through the means of the material mentioned (Fig. 7). I made many careful examinations and dissections of this bone in *Circus* before I was satisfied of what I saw, and that the condition existed as I have described it. In *Falco sparverius* the connection between these two separate parts of the pubic bone is through the finest imaginable bony bridge, that passes close under the margin of the lower ischiadic border, and so far as I have examined the Falcons it is always present in them, though sometimes almost of hair-like dimensions.

Professor Owen says: "The shortest pubis is seen in certain Eagles, in which it terminates after forming the lower boundary of the obturator foramen, its extremities there projecting freely, as in Fig. 23 (*d.* Side view of pelvis, Eagle), or being joined by ligament to the ischium, as in the Harpy Eagle, in which it is an inch in length, whilst the ilium is six inches long." ("Anat. of Verts," p. 36, Vol. II). I am sorry to say that at the present writing I have not the complete skeleton of an Eagle before me, and no pelvis of that bird. I would not be surprised to learn, however, that the skeleton that fell to the lot of this eminent anatomist to examine at the time he made the above statement was an imperfect one, and that the hinder three-fourths of the pubis on both sides was lost, a thing very likely to happen were they connected to the anterior portion by a delicate bridge of bone, or entirely disconnected as we find them in *Circus*. It may be that specimens of *Circus* will be taken where the fine bony, almost hair-like, connection will be seen to join these two parts of the pubis, but so far I have failed to find one, and I must believe that the condition as I have described it above is the normal and perhaps constant one. Taking into consideration the state of these things as they exist in *Falco sparverius* it is very easy to conceive how such a condition might come about as we see it in *Circus*—the fine ligamentous span simply no longer ossifies—as whatever the original necessity was for weakening the pubis at this point it has been eventually accomplished, and ossification is now no longer extended to that part of the pubic rod at all. The free hinder ends of these bones in *Circus* are now completely movable, as anyone can satisfy himself about by examining these parts in a freshly killed specimen.[1]

---

[1] Since writing the above I have detected this condition of the post-pubis in other Falconidæ, and the reader is referred to my remarks about it in *The Auk*, January, 1886, p. 133, where I give a figure showing how it also occurs in *Buteo borealis calurus*. Professor W. K Parker, F. R. S., tells me, too, in a valued letter I have from him, that this state of things also occurs in some of the Old World Falconidæ, and that in them the post-pubis is occasionally aborted, "which is a very interesting fact."

The twentieth and twenty-first vertebræ seen beneath the ilia have already been sufficiently described. Posterior to them on the ventral aspect of the pelvis a considerable swell takes place in the column to accomodate the sacral enlargement of the cord. This gradually contracts again at a point opposite the anterior borders of the acetabulæ.

The *twenty-second vertebra* throws up both parapophyses and transverse processes against the ilia. In this the next three succeeding vertebræ follow suit. This takes place at the narrowest part of the pelvis, and these processes are very stout here.

Fig. 10. Superior aspect of pelvis of *Circus*; same specimen as the last. Life size from nature.

In the *twenty-sixth* to the *twenty-ninth*, inclusive the short abutting processes cannot be seen upon direct ventral aspect. This is the region of the true "sacrum" and the foramina of exit for the sacral nerves are here double on either side, one opening being above another.

The *thirtieth*, *thirty-first* and *thirty-second vertebræ* have long parapophyses, which amalgamate at their outer extremities, where they form a powerful abutment for the pelvic walls at points opposite the acetabula. The pelvis of this Harrier is deep in all this region, that is, posterior to the twenty-fifth vertebra and including the three I have just mentioned.

The pelvic bones behind grasp but two more segments of the column, the *thirty-third* and *thirty-fourth* vertebræ. These much resemble the anterior coccygeal ones, especially the last one.

In the *coccyx* we find six vertebræ freely movable on each other, but with nothing peculiar about them. The fourth and fifth of this series have equal and at the same time the most far extending transverse processes. The width of the last (the sixth) is about equal to the first, and the last three have bifid hyapophyses. *Circus* has a very broad and lofty *pygostyle*, that in the adult bird shows but few traces of its original composition. Its anterior edge is sharp, while behind it is flattened and narrowly triangular with the base of the triangle below. I give a posterior view of this bone in my "Osteology of the *Cathartidæ*," in "Hayden's 12th Annual," p. 761, where it is compared with the bone as it occurs in other Hawks and Falcons. Say nothing of a very few, not weighty points of difference existing between the pelvis of *Circus* and the same bone as we find it in many of the Owls, we may state that, in general resemblance, the pelvis of this Harrier is far more like that of an Owl than it is like the pelvis of any of the true Falcons. The pubic bones of an Owl, however, are nearly of uniform calibre throughout, from acetabulum to extremity. (See my figure of *Asio wilsonianus* in Coues' "Key to N. A. Birds," 2d ed., p. 136).

In all of these birds the pelvis is a pneumatic bone, and in *Circus* this condition is partially extended to the first two free coccygeal vertebræ, but not beyond them.

In the *pectoral arch* we find that a *scapula* is broad and truncate posteriorly, with its apex drawn out into a spicular-form point. Its neck is thick and broad, being sub-elliptical upon section (Figs. 7 and 9); while on the articular surface it extends to the glenoid cavity, and is about half or a little more than that presented by the coracoid. Upon its under surface, close to the line of articulation with this latter bone, we find a circular pneumatic foramen, which is constant. This line of articulation runs out to the end of the scapular process of the coracoid, but beyond this the scapula is extended as a clavicular process which meets the head of the furcula (Fig. 9), thus closing in the tendinal canal. In my drawing of these bones in the "Osteology of the *Cathartidæ*," there is a slight separation at the point just referred to, which is correct. The proper relations of these bones in *Circus* are shown in Fig. 9 of the present memoir. In *Circus* all the thoracic pleurapophyses are overlapped by the scapula, except the last two pair, so we may judge from this that this bone is below the average length for birds, not reaching the anterior border of the pelvis.

One would hardly expect from an examination of the sternal bases of the coracoids that they decussated in their grooves, as these parts are apparently exactly alike in either bone. The inner angle is carried out as a sharp point while the outer is a stumpy process (Fig. 7). A strong muscular line marks the shaft anteriorily, especially at its lower part, the shaft itself being stout and subcylindrical at its middle third. Just below the inner end of the scapular process, we find on the side of the shaft a long shallow notch, which in life is spanned by a delicate ligament, thus converting the notch into a foramen. In many owls this foramen pierces the wing of the scapular process of the bone near the centre, as in *Speotyto*, where I found it transmitted a branch of that cervical nerve coming from between the twelfth and thirteenth cervical vertebræ. (Osteology of *Speotyto cunicularia hypogæa*—Hayden's 12th Annual.)

The scapular process of the coracoid has already been alluded to above when describing the scapula. It is comparatively very small and shows but little on direct inner view (Fig. 9). It holds the same position as seen in *Ibycter americanus*, *Micrastur semitorquatus*, *Buteo borealis* and others studied by Ridgway, and so strikingly compared in his "Outlines of a Natural Arrangement of the Falconidæ."

Upon the anterior aspect of the coracoid or really on the head of the bone, there is an elongated facet placed vertically and slightly raised above the surrounding parts, which articulates with a broad surface of similar form on the outer side of the expanded head of the clavical, this latter surface looks directly backwards, a special recess being made for it. The meeting of the two bones is extensive and very intimate, as I have elsewhere pointed out.

The rounded tuberous head of the coracoid rises but little above the broad surface of the anterior end of the clavicle, and this projection arches over a recess at its inner aspect in which is hidden large pneumatic foramina that communicate with the hollow shaft and other parts of the bone.

The *furcula* or the united *clavicles* are likewise highly pneumatic bones; the foramina that enter them being found upon the non-articulating surface, opposite the foramina just described as perforating the inner side of the head of the coracoid. When the two bones are *in situ*, these two surfaces form the anterior walls of a fossa that lies immediately beyond the "tendinal canal" and really a part of the same enclosure.

Above, the clavicles are broad and articulate with the sides of he heads of the coracoids, and the clavicular process of either scapula in a manner already described. Viewed from in front they present the extreme type of the U-shaped style of the bone, the internal periphery of the arch being nearly a semicircle. The bones are compressed from side to side, and diminish in breadth as they approach the point of union below.

Here the clavicles support a small tuberous hypocleidium, which, owing to the backward curvature of the fourchette, is about opposite the coracoidal beds on the sternum. A well developed *os humero scapulare* is supported in the usual manner at the back part of the shoulder joint. It is quite a characteristic of the raptorial as well as other groups of birds, and is of great service in increasing the osseous articular surface for the humerus.

*Of the Pectoral limb.—Circus* in common with many other diurnal raptores and Owls has but one pneumatic bone in the skeleton of its wing, and this is the humerus (Fig. 11). This bone is thoroughly permeated with air, and although of good size, it is very light indeed. The pneumatic fossa is of an elliptical outline, occupying its usual site, and at its base numerous pneumatic perforations occur. Over it curls the ulnar tuberosity, forming for it contracted margins on three sides, making the entrance smaller than the fossa inside.

The articular tuberosity for the glenoid cavity is spindle-shaped and not very extensive. A decided valley divides it from the ulnar tuberosity. Bending over towards the palmar aspect of the bone we observe a prominent radial crest. This extends from the upper end of the articular tuberosity, 3 centimeters down the shaft. In form it is a long isosceles triangle, with the angle above, and the base on the shaft, (the bone being alongside the body of the bird in a position of rest, the one it occupied as I describe it). Viewed from above in this position, the humerus has the usual long *ſ* form. Smooth and cylindrical, the middle third of the shaft presents nothing of special interest. Distally, it dilates as usual to support on its palmar aspect the radial and ulnar tubercles; a muscular tuberosity occurs above each of these for tendinal insertion. A broad, deep valley is behind the oblique and ulnar tubercle occupying the anconal and distal extremity of the bone, to guide the passage of tendons to the antibrachium.

The *radius* of *Circus* has a length of 11 centimeters, being a slender and nearly straight bone. Its head presents an elliptical, concave facet for the oblique tubercle of the humerus, of considerable size, while the facet for the ulna about its head is not so extensive. Just below this latter is the tuberosity for muscular insertion.

The distal end of the radius is somewhat expanded transversely, to allow room for the grooves for the passage of the tendons, and a small articulation for the base of the *os prominens*. Below occurs the usual facet for the *radiale*.

The olecranon of the *ulna* is fairly well-marked as a rounded tuberosity, extending some two or three millimetres beyond the circular and concave facet intended for the ulnar tubercle of the humerus. It also has the usual articular concavities for the oblique tubercle and the head of the radius. The shaft of the bone is nearly four times the bulk of the shaft of the radius in calibre ; it is cylindrical and but slightly curved, showing only very faintly the row of papillæ for the quill-butts of the secondaries, adown its length.

Nothing of marked importance presents itself for our examination at the distal extremity of the ulna of *Circus*. The bone has here the usual articular surfaces and tuberosities for radiale and ulnare. Several years ago I described the ossicle of the antibrachium, in the *Nuttall Ornithological Bulletin*, as it was found in *Circus*, and named it the *os prominens* referred to above. In that article I present a cut showing its relations to the neighboring bones and the insertion of the extensor patagii longus. (Oct. 1881). This ossicle had previously been noticed by Prof. A. Milne Edwards, in a Kestril in his "Essai sur Appareil Locomoteur des Oisseaux." Mivart in his "Lessons in Elemantary Anatomy" (p. 320) also gives a cut, (after A. Milne Edwards), showing its position in the wing of an Eagle (*Aquila fucosa*).

Later, (April, 1882), in the *Nuttall Ornithological Bulletin*, Mr. Frederic A. Lucas in his "Notes on the Os prominens," made some valuable additions to our knowledge of the subject, presenting a list of many Hawks and Owls in which it occurred, and gave excellent figures showing its position in *Bubo virginicanus*, *Otogyps Calvus*, and others. In the chapter on the Anatomy of Birds in the second edition of his "Key," I note that Professor Coues adopts the name I originally bestowed upon this sesamoid. It seems that a bone which attains the size it sometimes does in certain birds, ought to be entitled to a distinctive appellation.

The *metacarpus* of this Harrier is a little over six centimeters long. Its articular surface for the carpal segments is quite oblique, and the part which originally was the first metacarpal, now forms an unusually prominent and projecting process, slightly bent to the anconal side. Wedged in between the proximal end of the bone and the distal ends of ulna and radius are found the usual carpal ossicles the *ulnare* and *radiale*. They differ in no marked respect from the bones as found in nearly related forms, or in some of the Owls.

The *pollex* has but one phalanx awarded it, but this bone is broad and strong, presenting a considerable articular surface for the metacarpus. This phalanx may support a claw at its extremity. *Second digit* has two phalanges, the usual one with posterior border, and the lower, long pointed one. In the former the expansion alluded to is surrounded by a prominent raised margin, but the area it encloses is not perforated as in some birds. The little bone of *third digit* has a process developed upon its posterior edge, which I have noticed in other birds, and will have something to say about on a future occasion.

*Of the Pelvic Limb :* As in the upper extremity so we find it to be case here in this member,—it is only the *femur* of the several bones of the limb, that is pneumatic. A neat, elliptical orifice is found on the anterior surface of the bone about the middle of its upper third between the trochanterian ridge and the anterior muscular line. This is its usual site when present in birds, and here admits air throughout all parts of a comparatively large, but very light bone.

The head of this femur is rather small, and placed at a slight angle on the shaft. Above, it is well excavated for the ligamentum teres, between which concavity and the trochanterian ridge, here but slightly elevated, we find the broad articular surface for the antitrochanter of the pelvis. Its breadth increases as it recedes from the head of the bone, and is carried slightly over the summit of the ridge.

This bone is gently arched throughout its length, the concavity being upon its posterior aspect. Its shaft is very smooth, for the most part cylindrical on section, and but faintly shows the usual muscular lines adown its length ; the most distinct one passing from the fore part of the trochanterian ridge to the outside boundary of the inner condyle. At the anterior aspect of the lower third of the shaft, we observe that the ridges which are the beginnings of the condyles are parallel with each other until they disappear at the lower end of the bone. They are quite prominent and thus give rise to a well marked "rotular channel." A very noticeable thing about the femur of *Circus* is that the condyles are about on the same level at their lowest points. If the bone is held vertically against a plane surface tangent to these points, the axis of the shaft is very nearly perpendicular to the plane. This is by no means the rule with a great majority of birds, where the inner condyle is produced beyond the outer one. It is well shown in a specimen of the femur of *Geococcyx* before me.

The inter-condyloid fossa is broad and fairly divided from the popliteal depression by a low transverse bar. As usual, the outer condyle is vertically cleft behind to afford an articular cavity for the head of the fibula. The little tuberosities for muscular and ligamentous insertion about this end of the femur in *Circus* are well marked, and the foramen for the entrance of the medullary artery occupies its usual site on the posterior aspect of the shaft below the juncture of the upper and middle thirds.

In the skeleton of *Circus* as it is ordinarily prepared for study, the *tibia* and *fibula* are highly characteristic of the non-pneumatic class of bones, being dark, and for the most part of a deep amber color and greasy. The former is but little curved forward, as we sometimes see it, the shaft being very straight from any point of view (Figs. 12 and 14). Seen directly from above, the proximal articular surface for the condyles of the femur is nearly square (Fig. 14). The intercondylar convexity is but feebly pronounced, and the rotular crest of the bone rises but slightly above the general undulating articular surface. The apex of the entocnemial ridge points directly outwards, the opposite or procnemial rldge being developed as a crest parallel with the outer surface of the fibula, and produced some distance down the shaft of the bone. Below, and on the outer side of the shaft of the tibia, we find a long, well developed, fibular ridge, for the usual articulation of that bone. Further down the shaft its continuity is subcylindrical on section, at least as far as where it begins to become antero-posteriorly flattened above the condyles.

The usual oblique bony bridge for the retention of tendons is seen on the anterior aspect just above the condyles, and above it again the two tubercles, one on either side, for the attachment of the ligament that performs a similar function. Of these latter the inner is the higher on the shaft. The tibial condyles are nearly of a size, the outer one being produced the further up the shaft posteriorly. In this situation the articular surface merges across the inter-condyloid space. On the outer aspect of the inner condyle a hemispherical tubercle forms a striking object. *Circus* has but a single *patella*. This bone is of a cordate form with the rounded apex below, and a transversely truncate surface above. Posteriorily it is more convex than it is anteriorily, and it has a transverse diameter of five millimetres at it greatest width.

The *fibula* (Figs. 12 and 14) is laterally compressed above, the hinder part of its head extending backward over the shaft. It does not rise above the articular plane of the tibia, and only touches it above near its anterior and inner angle (Fig. 14). At 1.3 centimetres down its shaft it comes in contact with the fibular ridge of the tibia, opposite which it develops on the outer side of its shaft the usual tuberosity for the insertion of the tendon of the biceps. Its contact with the fibular ridge extends for two centimetres along the tibial shaft. Below this the fibula does not again come in contact with the latter until it passes its middle point from whence its needle-like dimensions may be traced in close contact with the main bone of the leg to the juncture of its middle and lower thirds.

The Marsh Harrier presents us with a very interesting form of a *tarso-metatarsus*, (Figs. 13, 15 and 16). Viewing its proximal extremity directly from above, (Fig. 15) we note that there are two distinct processes representing the "hypotarsus." The inner of these is the longer, and both have slightly dilated extremities. They are at right angles to the shaft, and separated from each other by an interval of four millimetres, the base of the intervening valley being roundly concave from side to side. The articular surface at the extremity of the tarso-metatarsus presents two well-marked depressions for the condyles of the tibia. They are separated in the middle line by a slight convexity. Upon direct front view this bone appears to be straight, but seeing it laterally shows it to be greatly curved from one end to the other, the concavity being along the front of the shaft. It is much scooped out anteriorly, just below the articular end (Fig. 13). This is continued a short distance down the shaft, trending towards the inner side. At its deepest part, above, the bone is pierced from before, backwards, by a single foramen. Below this, and rather to the outer side there is a small, elongated, though prominent tubercle. Running down the front of the shaft from a point on the periphery of the proximal end, opposite the middle of the external articular depression for the condyle of the tibia, to the middle of the outer trochleæ, there is a rounded and pronounced crest, much like the tibial crest in some vertebrates.

The outer aspect of this bone is broad and flat, being the broadest at mid-shaft. The inner, and at the same time in part the anterior aspect is likewise broad and flat, though carried to a sharp edge above on the inner aspect proper. This latter side is not as broad as the outer aspect. These two surfaces meet to form the anterior crest described above. From between the hypotarsial processes to the trochleæ, the posterior surface of the shaft is deeply and longitudinally grooved.

The margins of this excavation are sharp throughout their extent, being the posteior edges of the two surfaces described as the inner and anterior and the outer surfaces, above.

Upon the lower edge of the inner of these two margins we find a well marked elongated facet, intended for the articulation of a large-sized metatarsal. This latter bone is of considerable size in this Hawk, and its distal trochlear surface is very broad, being placed transversely on the bone. Above, it is so articulated as to allow of considerable freedom of movement, being attached to the tarso-metatarsus in the most usual manner by ligament. This distal end of the tarso-metatarsus, that bears the trochlear facets, is much expanded in a lateral direction, being gently convex from side to side, anteriorly where it shows the usual foramen for the anterior tibial artery. Behind, it shows an amount of concavity, from side to side, equal to the convexity of the anterior aspect. The trochlear processes for the pedal digits are separated by not very deep notches. Their lower surfaces are about in the same plane, the inner one perhaps being rather lower than either of the others, though not noticeably so at first sight.

The mid-trochleæ presents a deep median antero-posterior groove, not well marked in either of the others. In comparing the hypotarsial processes of the tarso-metatarsus as they occur in *Circus*, as I have endeavored to describe them, above, with the same processes as they are found upon the tarso-metatarsal of *Asio wilsonianus* and *Falco sparverius*, some interesting points become apparent. The arrangement in *Asio* is much the same as we find it in *Circus*, there being a single foraminal perforation, while the pedicle of the inner process in *Asio* is comparatively a little deeper from above, downwards. In the Sparrow Hawk, however, we are met by a very different state of things. Here we find the outer hypotarsial process shrunk up to the merest apology for such a thing, while the inner one holds a *mid-shaft* position, becomes a very prominent crest, which is carried down the posterior aspect of the bone for fully two-thirds of its length, gradually disappearing in the middle of its lower third. Above, it is pierced on either side of the crest by a foramen, these being placed nearly side by side, with the wall of the crest, just described, between them.

The proximal end of the first digit of *hallux* is broad and subcompressed ; the shaft of the bone is strong and stout—its upper aspect is rounded, while below it is flat and slightly grooved longitudinally. The trochlear surface is deeply scooped out in the median line, more especially underneath—an arrangement which allows the ungual phalanx to be thrown well towards the sole of the foot. Thus strongly flexed, this Harrier in common with other birds of prey can firmly hold the victims they seize, and even with ease drive their talons into their very flesh. The ungual phalanx of hallux is a powerful bone, curved throughout and sharp-pointed. When held in the position it has when the bird is standing, we observe the following points for examination at its proximal end, from above downwards. First, a single median process, the superior convex surface of which is continuous with the line of the upper border of the claw. This process has on its under side a raised median ridge for articulation with the superior groove of the trochlea of the first phalanx. It is produced in the median line at an open angle over a circular projection below it. Here we find on either side of the ridge a concavity, the whole forming the articular surface for the inferior side of the trochlea of the first phalanx. Projecting in the median line downwards and backwards, from beneath the parts just described, another prominent process is seen, for tendinal insertion. First phalanx of hallux measures 1.8 centimetres in length ; the chord of the claw measuring two centimetres taken from apex to tip of that process which is the superior one when the foot is in the position of standing.

The first phalanx of the inside toe is a chunky, and at first sight a very irregular shaped bone.

Above, it is convex from side to side and presents a small tubercle on its inner aspect. Longitudinally the superior surface is limited, the two articular facets nearly meeting. Its lower surface is powerfully grooved for the passage of the flexor tendons, and its proximal end is fashioned to articulate with the tarso-metatarsial trochleæ.

The articular surface intended for the proximal end of the phalanx beyond, occupies a space both above and below the end of the bone. It is surrounded by a raised marginal rim, which allows the succeeding phalanx scarcely any motion in the vertical plane, and these latter joints of the digits have it in no other.

The second phalanx of the inside digit very much resembles the first digit of hallux ; it is, however, a smaller bone, the same may be said of its claw, though we note that the curvature is less in the one under consideration.

The irregular first phalanx measures seven millimetres in longitudinal axis between parallel lines which touch its most distal and proximal points. Second phalanx measures 1.7 centimeters and the chord of the claw measured as in the first instance 1.9 centimeters.

The four phalanges of the middle digit in *Circus* all more or less resemble the typical style of the joint, *i. e.*, like first phalanx of hallux. From proximal to distal one, the first three measured 1.6, 0.8, and 1.5 centimeters respectively ; the chord of the claw being 1.6 centimeters.

Measured in the same manner the joints of the outside toe give 0.8, 0.5, and 0.45 centimeters, and the chord of this claw 1.3 centimeters.

In some respects these proportions of the phalanges agree with similar measurements made upon certain species of Owls, and in others with the Falcons, but this subject I propose to hold in reserve for another memoir, in which it will be fully treated, and properly illustrated.

Ossification occasionally extends, in *Circus*, to some of the tendons of the lower extremities, in subjects several years old. Among the Owls this condition is the rule. I have seen it in *Bubo*, *Strix*, *Asio*, *Surnia* and *Speotyto*.

The usual parts of the sense capsules also ossify, as the sclerotals of the eye, and the *columella auris* of the organ of hearing.

It was the writer's intention to present a table giving measurements in centimeters and fractions, of the various parts of the skeleton of *Circus ;* but upon second thought this idea was abandoned, as such measurements are of far more use and interest when compared with similar ones made upon the skeletons of allied forms. So this will be deferred for another occasion.

I complete this paper with a recapitulation of the principal characters of the skeleton in this Hawk.

### OSTEOLOGICAL CHARACTERS OF CIRCUS HUDSONIUS.

1. The nasal septum in the dried skull is not complete, there being a deficiency at its superior posterior angle.

2. The osseous nares are of an elliptical outline on either side, the major axis being in the same straight line with the imaginary one drawn between the anterior point of the fronto-lacrymal articulation and a point five millimetres above the apex of the superior mandible.

3. The maxillo-palatines are spongy bones, being attached to the nasals and nasal septum in the rhinal chamber, merging into each other anteriorly only, being produced posteriorly, parallel and separate, as far as an imaginary line joining the antero-inferior angles of the ethmoidal wings.

4. A circular foramen exists on either side, immediately beyond the maxillo-palatine plate of the maxillary.

5. The vomer is a narrow plate of bone, curving upwards, then forwards, to terminate in a free-pointed extremity. More than its half lies between the maxillo-palatines. Behind, it is anchylosed with the palatines.

6. The interorbital septum has an elliptical fenestra in it of some size.

7. The canal for the passage of the olfactory nerve is double.

8. The larcrymals are freely articulated in the adult; and have no additional pieces at their outer extremities.

The basi-sphenoidal processes are present but rudimentary, not reaching the pterygoids.

10. The outer posterior angles of the palatines are rounded, and for the most part these bones lie in the horizontal plane.

11. The mandible is without a ramal vacuity. (Negative character.)

12. The axial skeleton contains forty (40) vertebræ; the first pair of free ribs are attached to the thirteenth; the dorsal vertebræ are freely movable upon one another; the twentieth vertebra is the first one that anchyloses with the pelvis; the thirty-sixth is the anterior free coccygeal vertebra; there are six (6) free coccygeal vertebræ and a large pygostyle; there are nine (9) pairs of ribs in all, the first two pair and the last one are without unciform appendages; the first two pair are free; the last two pair articulate with the anchylosed vertebræ beneath the ilia; seven pairs, intermediate, articulate with the sternum through hæmapophyses.

15. The pre-acetabular surface of the pelvis is double the extent of the post-acetabular; the ilio-neural canals are sealed over; the anterior fourth of the pubis closes in the obturator foramen, the hinder three-fourths of this bone is free, attached only to the ischium by ligament; a considerable space exists between the two portions.

14. The coracoidal grooves of the sternum decussate above the trihedral manubrium; the xiphoidal extremity of this bone may show one or two foramina in it, on either side, and its border is gently convex forwards.

15. The scapular process of the coracoid does not reach the clavicle. (Negative character.)

16. The humerus is the only pneumatic bone of the wing; there is a mid-apical summit to its radial crest; the ulna is 11.5 centimeters long; there is an os prominens present over the carpus; the digits of manus are devoid of claws, though pollex may possess one.

17. The femur is the only pneumatic bone of the pelvic extremity; the lowest points of its condyles are in the same plane to which the axis of the shaft is perpendicular; the patella is single; the tibia is one centimetre shorter than the ulna, it has the bony bridge below to confine the extensor tendons; the hypotarsus of the tarso-metatarsus consists of two separate processes, neither are extended down the shaft; the first metatarsal is a free and large bone, and the arrangement of the phalanges of the digits of pes is upon the most common plan; the long axis of the proximal phalanx of the inside toe is less than half as long as the long axis of the phalanx that next succeeds it.

## Figures and Tables

**Fig. 1. Fig. 2. Fig. 3. Fig. 4. f1:**
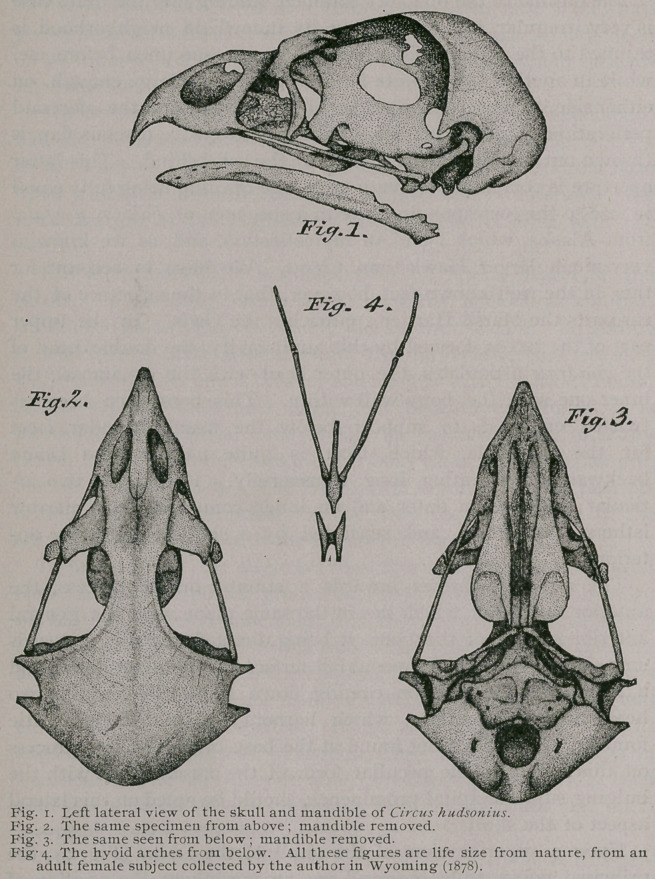


**Fig. 5. f2:**
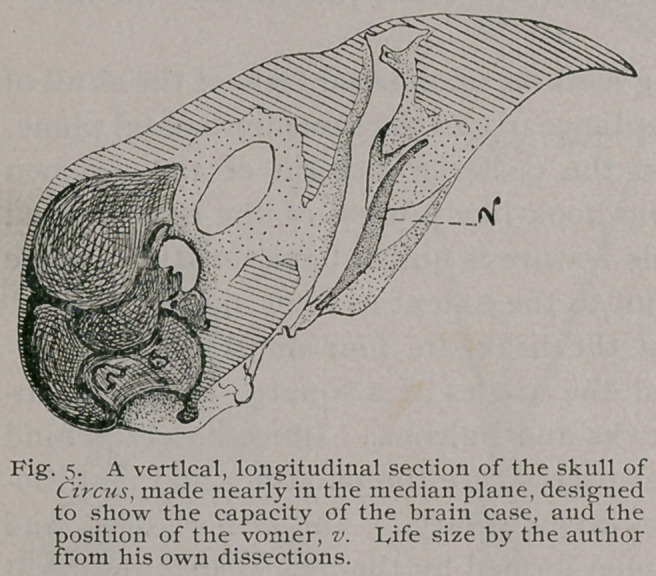


**Fig. 6. f3:**
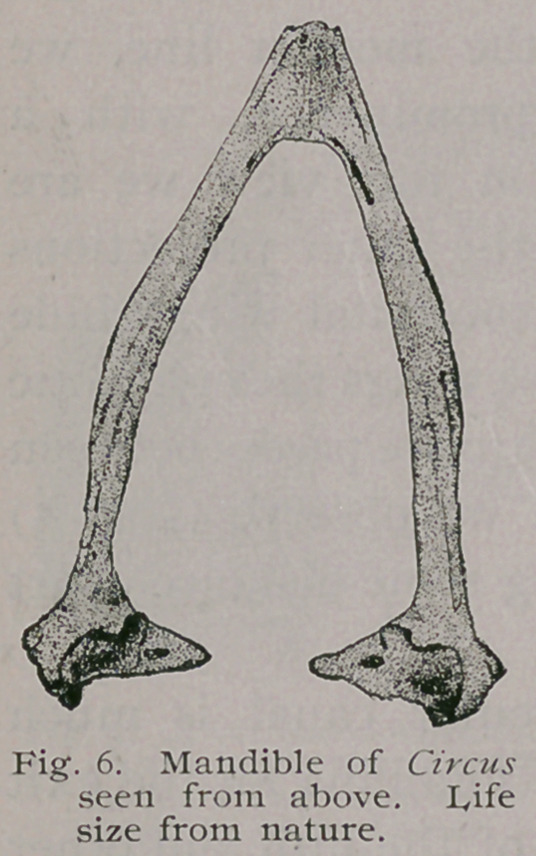


**Fig. 7. f4:**
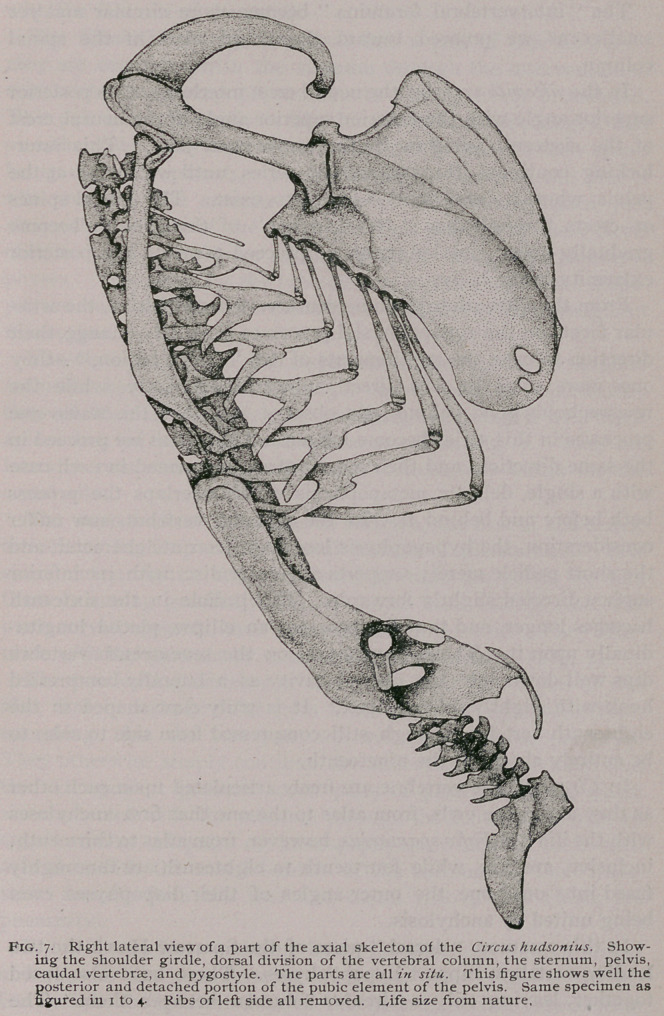


**Fig. 8. Fig. 9. f5:**
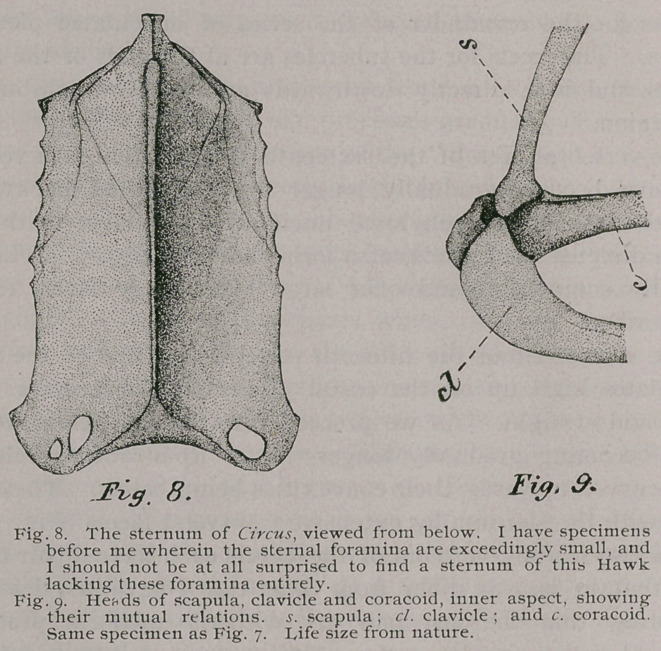


**Fig. 10. f6:**
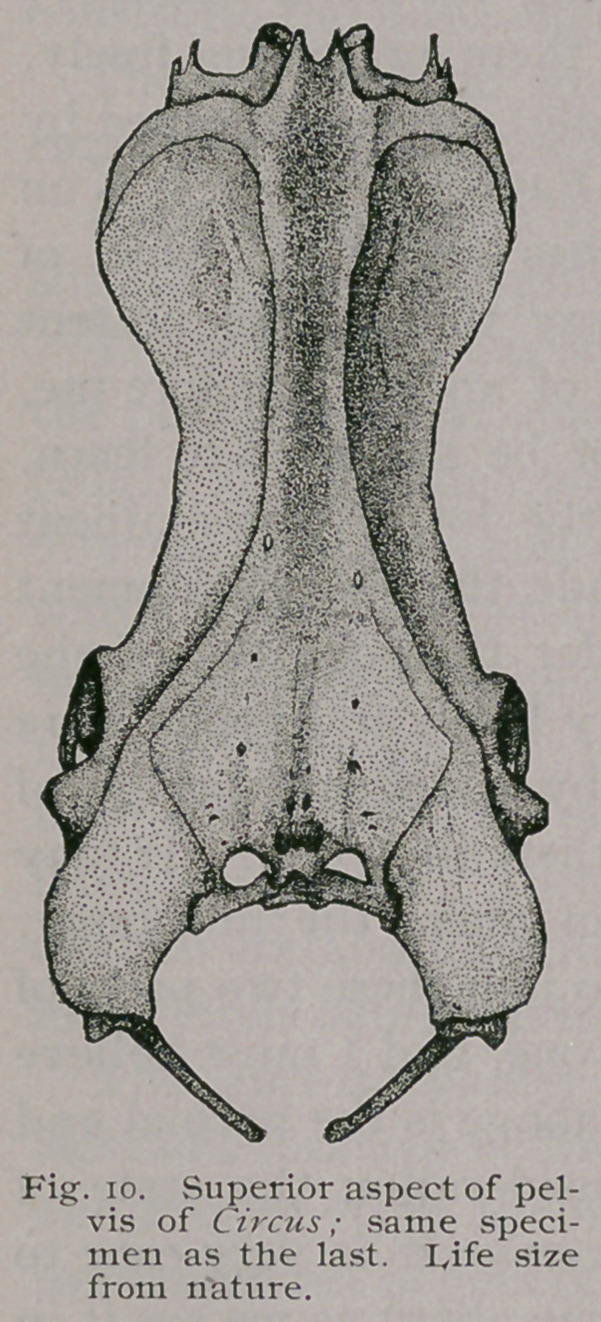


**Fig. 11. Fig. 12. Fig. 13. Fig. 14. Fig. 15. Fig. 16. Fig. 17. f7:**